# Sepsis-Related Lung Injury and the Complication of Extrapulmonary Pneumococcal Pneumonia

**DOI:** 10.3390/diseases12040072

**Published:** 2024-04-03

**Authors:** Samuel Darkwah, Fleischer C. N. Kotey, John Ahenkorah, Kevin Kofi Adutwum-Ofosu, Eric S. Donkor

**Affiliations:** 1 Department of Medical Microbiology, University of Ghana Medical School, Accra P.O. Box KB 4236, Ghana; fcnkotey@flerholiferesearch.com (F.C.N.K.); esampane-donkor@ug.edu.gh (E.S.D.); 2 Department of Anatomy, University of Ghana Medical School, Accra P.O. Box KB 4236, Ghana; jahenkorah@ug.edu.gh (J.A.); kadutwum-ofosu@ug.edu.gh (K.K.A.-O.)

**Keywords:** *Streptococcus pneumoniae*, sepsis, dendritic cells, pneumonia, inflammation, lung injury, critical care, infectious diseases, ALI, PavA

## Abstract

Globally, sepsis and pneumonia account for significant mortality and morbidity. A complex interplay of immune-molecular pathways underlies both sepsis and pneumonia, resulting in similar and overlapping disease characteristics. Sepsis could result from unmanaged pneumonia. Similarly, sepsis patients have pneumonia as a common complication in the intensive care unit. A significant percentage of pneumonia is misdiagnosed as septic shock. Therefore, our knowledge of the clinical relationship between pneumonia and sepsis is imperative to the proper management of these syndromes. Regarding pathogenesis and etiology, pneumococcus is one of the leading pathogens implicated in both pneumonia and sepsis syndromes. Growing evidence suggests that pneumococcal pneumonia can potentially disseminate and consequently induce systemic inflammation and severe sepsis. Streptococcus pneumoniae could potentially exploit the function of dendritic cells (DCs) to facilitate bacterial dissemination. This highlights the importance of pathogen-immune cell crosstalk in the pathophysiology of sepsis and pneumonia. The role of DCs in pneumococcal infections and sepsis is not well understood. Therefore, studying the immunologic crosstalk between pneumococcus and host immune mediators is crucial to elucidating the pathophysiology of pneumonia-induced lung injury and sepsis. This knowledge would help mitigate clinical diagnosis and management challenges.

## 1. Introduction

Pneumonia and sepsis are two critical, life-threatening health syndromes with a far-reaching global impact. Significant economic and public health burdens associated with these conditions persist, despite years of efforts to develop and advance medical interventions [1,2]. The term ‘sepsis’ defines a complex clinical syndrome characterized by a dysregulation of the body’s immune response to an infection. Pneumonia, on the other hand, is defined as an inflammation of the lung due to an infection, causing air sacs to be filled with fluid (pus), consequently obstructing gaseous exchange. 

Infections and inflammation are key clinical features that underlie sepsis and pneumonia; thus, several clinical presentations run commonly across these two complications. As a result, severe sepsis and septic shock can often be misdiagnosed as pneumonia—a flaw in diagnosis that raises further health concerns, such as inappropriate medical interventions. Interestingly, about 29% of patients with pneumonia were observed to be patients wrongly diagnosed with septic shock [3]. Clinically, severe sepsis is identified as a critical condition complicating pneumonia, and is associated with a lengthened hospital stay, worse prognosis, increased healthcare cost, and mortality [4,5]. Patients in critical care units, such as severe sepsis and septic shock patients, are a major risk group susceptible to nosocomial infections. Pneumonia ranks among the most common nosocomial infections complicating the health of critically ill patients. Sepsis patients with septic shock could suffer acute hypoxemic respiratory failure [6] and may require intubation or some invasive mechanical ventilation. Since ventilator-associated pneumonia commonly develops in mechanically ventilated patients [7], sepsis patients in shock are at an increased risk of pneumonia. Understanding the similarities, differences, and overlaps of sepsis and pneumonia is essential for the proper management of these critical conditions.

In this review, we discuss the pathogenesis, etiology, and epidemiology of sepsis as well as pneumonia. We review the clinical relationship between pneumonia and sepsis: (i) sepsis-induced acute lung injury; (ii) pneumonia and the risk of sepsis. A closing section titled ‘The pneumococcus and septic inflammation’ attempts to highlight the relationship between pneumococcus (an important bacteria implicated in both pneumonia and sepsis) and systemic inflammation. We discuss the crosstalk between pneumococcus and relevant immune cells, and how these interactions, along with other immune mediators, potentially influence the progression of pneumonia and sepsis.

## 2. Pneumonia and Sepsis (Pathogenesis, Etiology, and Epidemiology) 

### 2.1. Pneumonia

Pneumonia can primarily be defined as an infection-induced inflammation of the lung parenchyma. It is an umbrella term used in healthcare to describe a group of syndromes caused by a myriad of organism types, leading to various manifestations and sequelae of pulmonary malady. This implies that the term pneumonia does not denote a specific disease, but rather a persistent burden of disease [8,9,10]. Although pneumonia is not always fatal, severe cases are rampant, with a significant number of risk groups identified to require intensive medical care following its onset [10,11]. Individuals with chronic medical conditions such as diabetes, chronic obstructive pulmonary disease, smoking-related morbidities, and those who may suffer from acute events (e.g. trauma, and air pollution) are particularly susceptible to pneumonia [12]. 

A physiologically delicate but complex balance exists between the microbiome of the lower respiratory tract and the host immune mechanisms. A disturbance in this balance is implicated in the onset of inflammation of the lung parenchyma. The development of pneumonia, therefore, will involve an invasion of the lower respiratory tract by an infective microorganism, leading to a concomitant overwhelming host inflammatory response against the offending microorganism’s virulence, inoculum size, or both. The severity of an established case of pneumonia can primarily be warranted by two processes in the host biology: immune-resistant pathways responsible for curbing microbial growth and invasion, and host tissue resilience to ameliorate the inflammatory burden in the lung [12,13]. 

Pneumonia can be classified into three groups, according to the American Thoracic Society (Table 1) [14,15,16], and has four distinct pathological stages broadly described under two histological domains, namely, lobar pneumonia and lobular/bronchopneumonia (Table 2) [8,17,18]. Bronchopneumonia is characterized by patches of localized inflammation around the bronchi in one or more lobes of the lung. Lobar pneumonia, on the other hand, is an evolution of pathological changes involving an entire lobe of the lung. 

Several microorganisms have been identified to underlie the development of pneumonia. As a result, the etiology of pneumonia in clinical practice is not confined to one agent. A significant number of publications have confirmed that there is a relatively low proportion of patients with pneumonia of a single cause [19]. Thus, the etiology of pneumonia can better be described under the three classes of pneumonia (Table 1).

**Table 1:** Definition and classification of pneumonia.

Class of Pneumonia	Definition/Characteristics	Common Pathogens Associated
Community-Acquired Pneumonia (CAP)	All pneumonia acquired in a community setting, i.e., outside hospital confines. Includes pneumonia acquired at assisted-living facilities, rehabilitation centers, and other healthcare facilities.	Bacteria: *S. pneumoniae*, *H. influenzae*, *S. aureus*, *Moraxella catarrhalis*, Group A *Streptococcus*. Causes of ‘atypical’ disease include *Mycoplasma pneumoniae*, *Chlamydia*, *Legionella* [20,21,22,23]. Viruses: Respiratory syncytial virus (RSV), Adenoviruses, Influenza virus, and Parainfluenza virus. Fungi: *Blastomyces*, *Histoplasma*, *Coccidioides* [24].
Hospital-Acquired Pneumonia (HAP)	Acquired within 48 h following a person’s admission in a hospital setting only as an inpatient who has not been incubating an infection at the time of admission.	50–80% are associated with Gram-negative bacteria (*Enterobacteriaceae: K. pneumoniae*, *E. coli*, *Enterobacter* sp., *Proteus* sp.), *H. Influenzae*, *P. aeruginosa*, *A. baumannii* [25,26]. Gram-positive bacteria (*S. aureus*, *Streptococcus* sp.) [27,28,29].
Ventilator-Associated Pneumonia (VAP)	All pneumonia acquired 48 h following endotracheal incubation Associated with polymicrobial etiology	

**Table 2:** The pathological stages of pneumonia.

	Name	Characteristics
Stage 1	Congestion	Vascular engorgement, accumulation of alveolar fluid rich in the offending infectious pathogens, heavy and engorged appearance to the lung tissue.
Stage 2	Red-hepatization	Substantive infiltration of blood cells (specifically RBCs and neutrophils) and fibrin into the alveolar fluid, firm and deep red gross appearance of the lung, resembling the liver.
Stage 3	Grey-hepatization	Greyish lobe appearance owing to the presence of fibrino-purulent exudates and broken-down RBCs.
Stage 4	Resolution	Terminal event characterized by the clearing of exudates by resident innate immune mob cells (macrophages) and the potential formation of residual scar tissue (depending on severity).

Recent studies have identified these stages of pneumonia as more of a side-by-side occurrence rather than an ordered and strict sequential stage of an affected lobe [19].

### 2.2. Sepsis

Sepsis, like pneumonia, can be described as a syndrome of pathophysiological and biochemical abnormalities stemming from infection [30]. Sepsis is a complex, life-threatening pathology that involves the dysregulation of host immune responses and dysfunction of a variety of immune cells, usually to infection [31]. Septic inflammation and its associated immune dysregulation often lead to impairments and damage of multiple vital organs when not treated urgently [31]. 

The onset of sepsis is classically characterized by an overwhelming outburst of inflammatory responses, i.e., the local and systemic storming of potent inflammatory mediators. Owing to this, earlier theories proposed that mortalities from sepsis were a direct consequence of the early hyperinflammatory phase of sepsis, warranting therapeutic strategies aimed at alleviating inflammation using anti-inflammatory mediators [32,33,34]. The intense trigger of the innate immune response, known as a ‘cytokine storm’, involves a series of interaction and activation processes that occur in immune cells via the detection of both host and microbial complexes (e.g., Damage-Associated Molecular Patterns—DAMPs, Pathogen-Associated Molecular Patterns—PAMPs, and Lipopolysaccharides—LPSs). These processes up-regulate the expression of inflammation-related genes, and the subsequent release of inflammatory mediators into circulation [35,36]. Inflammatory cytokines such as interleukin (IL)-6, interleukin (IL)-1, and tumor necrosis factor (TNF)-alpha are mediators contributing to the early cytokine storm seen in sepsis [30]. Recent findings have identified delayed immunosuppressive events among sepsis survivors as a significant contributor to overall sepsis mortality [37,38]. Current theories propose that the pathophysiology of sepsis includes a compensatory yet concurrent immunosuppressive inflammatory phase, which tends to persist after recovery from the early hyperinflammatory phase [39,40,41]. The majority of sepsis patients with a protracted illness often have an increased risk of microbial infections, usually with opportunistic pathogens and increased deaths from secondary infections, particularly pneumonia [42]. Although the immunological microenvironment of sepsis and septic shock is yet to be fully elucidated, sepsis immunotherapy targeted at delivering immunomodulation drugs holds promise. Therapeutic strategies now target both sepsis-induced immune paralysis of various components of the host immune responses, as well as early hyperinflammatory stages (cytokine storm) [43,44,45]. Sepsis does not only involve inflammatory and immune disorders but also abnormalities in coagulation, metabolism, and neurologic and endocrine networks. Notably, the pathogenesis of sepsis spans complex changes in multiple organs and tissue structures, down to the cellular and molecular levels [30].

Chronic infections constitute a key risk factor in the development of sepsis and septic shock. Lung infections (pneumonia), urinary tract infections, and abdominal infections predominantly make up the infection-based risk for sepsis. Other infections increasing the risk of sepsis include HIV and non-healing dermal wounds [40]. Bacteria are the most implicated among microorganisms causing sepsis; thus, approximately 62% and 47% of critically ill patients have blood cultures positive for Gram-negative and Gram-positive bacteria, respectively [46]. Bacterial pathogens implicated in septic shock have gradually skewed away from vaccine-preventable pathogens to *Staphylococcus aureus.* To illustrate, a study in the USA identified *Staphylococcus aureus* as the most common infective organism, causing about 18% of infections among children with severe sepsis [47,48]. The extensive use of antibiotics in treating infections among the critically ill has also contributed to the surge in sepsis caused by methicillin-resistant *Staphylococcus aureus* (MRSA) [49,50]. Other Gram-positive bacteria isolated from blood cultures of severe sepsis patients include *Streptococcus* spp. and *Enterococcus* spp. Common Gram-negative bacteria that account for sepsis include *Pseudomonas* spp., *Escherichia coli*, and *Klebsiella* spp. [51]. Aside bacterial infections, viral infections, and fungal infections, as well as non-infectious causalities such as impaired immunity and trauma, have also been reported as causes of sepsis [52,53]. Some common viruses implicated in sepsis include respiratory syncytial virus (RSV), hepatitis viruses, influenza type A and type B, coronaviruses (including SARS-CoV-2), herpes simplex virus (HSV), human metapneumovirus, adenovirus, and several viruses of hemorrhagic fevers [54,55]. Among fungal infections, invasive candidiasis accounts for about 70% to 90% of cases, and approximately 5% of severe sepsis and septic shock are caused by *Candida* spp.

The assessment of bloodstream and systemic infections is an important component of the diagnosis and management of sepsis and septic shock. Thus, the role of microbial detection methods cannot be overemphasized. Blood culture remains the most reliable and appropriate method for the diagnosis of bloodstream infections, particularly bacteremia and fungemia [56]. Modern automated blood culture incubation systems with high specificity and sensitivity are currently available for clinical diagnosis of bloodstream infections. Some commercially available automated culture systems include the BD BACTEC (BD Diagnostics, Maryland, USA), BacT/ALERT 3D (bioMérieux, North Carolina, USA), and VersaTREK (ThermoFisher Scientific Inc., Massachusetts, USA) [57]. 

### 2.3. Epidemiology of Pneumonia and Sepsis

In developed countries such as the USA, the incidence of pneumonia is about 25 cases per 10,000 adults, annually. High rates of pneumonia are preferentially exhibited among aged populations (63 cases per 10,000 adults for ages between 65 and 79 years and 164.3 cases per 10,000 adults for those aged 80 years or older) [11]. The incidence reported on CAP in Europe has a wide variation, ranging between 20.6 cases per 10,000 adults to 79.9 cases per 10,000 adults [58,59]. Epidemiological reports from developing countries reflect similar patterns of incidence; however, recent reports suggest higher incidence in the developing populations of South America [60,61]. With regards to mortality, lower respiratory tract infection is the second-most common cause of death according to a Global Burden of Disease Study in 2013, accounting for about 6% of deaths, and is considered the primary infective cause of death [2,62]. According to the World Health Organization (WHO), CAP is frequent and fatal, accounting for approximately 3 million deaths per annum worldwide [62]. UNICEF, in 2017, reported that pneumonia globally tops the chart of leading causes of infant mortality, accounting for 16% of 5.6 million deaths of children under 5 years old [63]. Global infant cases of pneumonia hover around 1400 cases per 100,000 children, or 1 case per 71 children per annum. According to UNICEF, the greatest incidence of neonatal pneumonia occurs in South Asia (2500 cases per 100,000 children) and West and Central Africa (1620 cases per 100,000 children) [64]. Most of these deaths occur in low-income and developing countries [65]. CAP is responsible for approximately 102,000 deaths and 23,000 deaths every year in the USA and Europe, respectively [66,67], with higher rates reported in low-income regions and developing countries [68]. The case-fatality rate of nosocomial or hospital-acquired pneumonia (HAP), which includes ventilator-associated pneumonia (VAP), is thought to be higher than that of CAP [69]. A recent report from Taiwan estimates the incidence rate for VAP among mechanically ventilated patients to be between 9% and 27%. However, with the increasing menace of microbial resistance in the hospital environment, case-fatalities associated with VAP are projected to be high significant mortality rates, ranging between 20% and 50% [16,70,71].

Sepsis is a very important global health incidence and is thought to be biphasic, meaning incidence peaks among early childhood populations and again in elderly adults. Sepsis and its related mortality are a concern with varying degrees of impact across various regions of the world [1]. From 2003 to 2015, the global estimate of the incidence of sepsis stood at approximately 43.7 cases per 10,000 person-years [1]. According to reports from a recent global study, there were 49 million cases of sepsis and 11 million sepsis-related deaths in 2017, which accounted for about 20% of all deaths throughout the year [72]. Severe sepsis is the most common cause of death among the critically ill in the non-coronary intensive care unit and is considered the leading cause of death in the United States [73]. Evidently, older people are more likely to develop sepsis than younger populations, owing to age-related factors such as the increased rates of comorbidities among the aged, age-related immune incompetence, and increased colonization with Gram-negative organisms in patients with records of long-term hospitalization [74,75,76]. Globally, most of the patients with sepsis in the ICU are above the age of 50 years, with 12% of such patients being over 80 years. Consequently, the risk of death from sepsis and hospital mortalities is much higher for patients above 80 years of age (an increased risk percentage that almost doubles that of patients aged below 50 years) [77]. In the last two decades, the case fatality for severe sepsis has declined, owing to advances in supportive care in the ICU [78]. Neonatal sepsis poses a great public health and economic concern in sub-Saharan Africa. According to the 2020 WHO Global Report on the epidemiology and burden of sepsis, children under 5 years of age accounted for about 20 million of all estimated sepsis cases worldwide, with an estimated 15% of all neonatal deaths attributed to sepsis in 2018. The overall incidence of neonatal sepsis is highest in low-income countries, with the highest incidence of neonatal sepsis occurring in pre-term and low-birth-weight infants. The incidence of neonatal sepsis within communities in middle-to-low-income regions is estimated to range between 5.5 cases per 1000 live births (based on positive blood culture) and 170 cases per 1000 live births (based on clinical diagnosis) [79]. In the year 2010, an estimated 1.7 million cases and 270,000 associated deaths were reported for neonatal sepsis [80]. A recent study reports a more conservative estimate of about 355,500 to 605,750 annual cases of and 177,500 to 302,870 associated deaths from neonatal sepsis for the year 2014, with an estimated economic burden ranging from USD 10 billion to 469 billion per annum [81]. Sepsis is costly and poses a great economic burden. The estimated average hospital-wide cost of sepsis in high-income countries is more than USD 32,000 per patient [82]. It is estimated that 40% of sepsis patients are re-hospitalized within 90 days after their discharge from a healthcare facility [83]. Not only does this increase the burden of sepsis on patients, but also aggravates pertinent challenges in healthcare and delivery systems.

## 3. Sepsis-Induced Acute Lung Injury

### 3.1. Acute Lung Injury

Acute lung injury (ALI) is a common, yet critical and lethal medical condition. It is a term that encompasses ailments characterized by acute, severe hypoxia not due to left atrial hypertension nor a continuum of clinical and radiographic pulmonary changes. In the mid-1990s, Ashbaugh et al. described a devastating and resistant syndrome prevalent among patients with respiratory failure. This syndrome was characterized by an acute onset of hypoxemia, tachypnea, loss of lung compliance, and X-ray evidence of diffuse alveolar infiltration following a variety of stimuli [84]. The syndrome is what is currently known as acute respiratory distress syndrome (ARDS); ARDS now describes a more severe form of ALI [85]. The 1967 definition of ARDS was revised many times over the past few decades. The American–European Consensus Conference postulated four classification criteria and an improved, diagnostic-oriented definition for ARDS in 1994. These criteria were (a) an acute onset of symptoms after a known risk factor with a maximum within a week; (b) severe hypoxemia resistant to oxygen therapy, with a more severe form of respiratory insufficiency defined by a ratio between arterial partial pressure of oxygen and fraction of inspired oxygen (PaO_2_/FiO_2_) ≤ 200 mm Hg (26.7 kPa) named “ARDS”, and a milder form of this syndrome with PaO_2_/FiO_2_ 200–300 mm Hg (40 kPa) named “acute lung injury (ALI)”; (c) diffuse bilateral infiltrates on chest X-ray; (d) absence of cardiogenic pulmonary edema verified by wedge pressure in the pulmonary artery ≤ 18 mm Hg or no evidence of left atrial hypertension [86]. More current criteria, known as the Berlin definition, were published in 2012. This projects three criteria for ARDS (mild, moderate, and severe, based on the degree of hypoxemia) that provided a relatively better predictive validity of mortality compared with the earlier criteria proposed by the American–European Consensus Conference. The Berlin definition describes ARDS as ‘acute hypoxemia, a ratio of partial pressure of atrial oxygen to the fraction of inspired oxygen less than or equal to 300 mm Hg on positive end-expiratory pressure greater than or equal to 5 cm H_2_O, together with bilateral infiltration on radiology that is not otherwise explained fully by fluid overload or cardiac failure.’ [87,88].

### 3.2. Risk Factors for the Development of ALI

ALI results from a direct insult to the lungs or can develop as a secondary complication to primary factors such as infection or inflammatory response. Several such inflammatory factors account for the development of ALI. Among these factors, sepsis, particularly that of pulmonary origin (implicated in approximately a third of cases), ranks as the most common risk factor, with pneumonia being the most common predisposing condition for the development of ALI [89]. Primary causes for ALI and ARDS involve complications such as aspiration of gastric contents, pulmonary contusion, ventilator-induced lung injury, inhalation injury, and pneumonia. ALI may also result as a secondary consequence of lung injuries from blood transfusion practices, traumatic brain injury, pancreatitis, sepsis, and shock [84,90,91].

Increased susceptibility to ARDS has been identified in several studies to be associated with a myriad of comorbidities and exposures. These include smoking cigarettes, excessive alcohol intake, and air pollution [92,93]. Gender and racial disparities influencing the development and mortality associated with ALI/ARDS have been reported in several studies. Studies suggest that mortality differs by patients’ race and/or ethnicity; however, underlying reasons remain poorly understood. A study in 2012 reported on an in-hospital-based cohort revealed that the development of ALI is a more likely occurrence in male patients (6.9% for men versus 4.7% for women). Additionally, ‘Black’ patients were significantly less likely to develop ALI than ‘White’ patients, although risk factors such as pneumonia and sepsis were more frequent in the former [94]. African-American men in the United States reportedly have the highest rate of ARDS-related mortalities compared with every other subgroup of men, with a mean annual mortality rate of 12.8 deaths per 100,000 African-American men [95].

### 3.3. ALI as a Secondary Complication to Sepsis

Although sepsis is well known to potentially complicate ALI, it is also a major cause for the development of ALI and one of the leading causes of death among sepsis patients [96]. The ill effects of systemic inflammation associated with sepsis greatly affect the lungs and thus, as much as 40% of septic patients tend to develop ALI [86,97]. The onset of ALI/ARDS following sepsis among critically ill patients is estimated to usually occur within 48 h after hospital admission. Cellular and molecular mechanisms underlying the relationship between inflammatory events during sepsis and the development of ALI/ARDS have been well studied, however, more understanding of these mechanisms is required for the development of more advanced therapeutic strategies for treating and managing sepsis-induced ARDS [98]. Primarily, the immune system of patients with sepsis is dysregulated; therefore, unchecked pro-inflammatory signaling cascades accelerate the dysfunction of vascular endothelium. Consequently, these promote the excess influx of inflammatory cells (neutrophils, monocytes, macrophages, and lymphocytes). In such severe systemic inflammatory cycles, there is potential injury to the pulmonary microvasculature, which progressively results in ARDS. Disrupted permeability of pulmonary vascular endothelium and the loss of alveoli epithelial cells as a result of sepsis-induced apoptosis and necrosis allow for the filling of alveoli with plasma exudate. Additionally, the increased exudate in alveolar spaces causes edema in the alveolar and the formation of a hyaline membrane [90,99]. Patients with sepsis-related ARDS present with more significant dyspnea (lower PaO_2_/FiO_2_ ratios than do patients with non-sepsis-related ARDS) [100]. Patients who develop sepsis-associated ARDS are more likely to have a prolonged recovery from a lung injury, with a relatively low success rate of withdrawal from mechanical ventilation and a slower rate of extubation [101]. 

### 3.4. Sepsis-Induced Immunosuppression and the Susceptibility to Secondary Pneumonia

Despite the aggressively uncontrolled and sustained inflammatory response observed among septic and critically ill patients, a severe form of sepsis can be characterized by late-stage immune paralysis that is much associated with late mortality. Such immune dysfunction predisposes sepsis patients to potentially life-threatening secondary bacterial, viral, and fungal infections [102]. A compensatory recovery from hyperinflammation to a hypo-inflammatory state to limit organ dysfunction and shock during the events of sepsis is usually dominated by a late period of immune dysfunction. This dysfunction features anergy-impaired immune response to antigens with diminishing cytokine release from T-lymphocytes, a shift in the production of inflammatory mediators from inflammatory to anti-inflammatory sets, death of potent immune cells, and the expansion of paralyzed or exhausted immune cell phenotypes [32]. Among secondary infections implicated in severe sepsis-induced immune dysregulation, Ventilator-Associated Pneumonia (VAP) ranks high in terms of mortality and morbidity [103]. VAP among severe septic patients is usually caused by infections from common oropharyngeal commensals such as *Streptococcus* spp. and *Staphylococcus* spp. Resistant strains of *Pseudomonas* spp. and *Staphylococcus aureus* (MRSA) are also implicated in VAP following severe sepsis in susceptible patients, making treatment more difficult with a worse prognosis. The height of immunosuppression induced by severe sepsis has been shown to correlate with the occurrence and severity of VAP. A study revealed that increased susceptibility to nosocomial pneumonia in children with multiple organ dysfunction syndrome was associated with the persistence of immune paralysis [104,105]. Increased risk of nosocomial pneumonia has been reportedly linked with the reactivation of latent viruses such as cytomegalovirus (CMV) and herpes simplex virus (HSV) among critically ill patients. This has been explained by the impairment of innate and adaptive immune defects resulting from severe sepsis-induced apoptosis [106]. 

## 4. Pneumonia and the Risk of Sepsis

### 4.1. Pneumonia as a Cause of Sepsis

Sepsis remains a common complication of CAP, with at least a third of CAP patients presenting to the hospital with severe sepsis [107]. A study reported that 38 out of every 100 patients hospitalized for CAP had already developed community-onset severe sepsis on admission [108], reaching as high as 71% among very old patients presenting with severe CAP [109]. Septic shock is reportedly the main risk factor for mortalities associated with severe CAP. Such high mortality rates are associated with sepsis developing as a consequence of pneumonia due to frequent multi-organ dysfunctions [27,110]. Similarly, nosocomial pneumonia is often complicated by secondary sepsis. Approximately 36% of adult patients with non-ventilator HAP develop sepsis, a proportion comparable to that seen in patients with ventilator-associated HAP who develop sepsis [111,112]. A retrospective cohort study has reported that the majority of hospitalized patients diagnosed with CAP of viral causes present with sepsis; over 60% of viral, non-bacterial CAP patients developed viral sepsis. The most common viruses associated with viral sepsis secondary to viral CAP were found to be influenza virus, followed by rhinovirus, parainfluenza virus, respiratory syncytial virus, adenovirus, and coronavirus (including the recent, novel MERS-CoV and SARS-CoV) [113].

### 4.2. Pneumococcal Pneumonia

*Streptococcus pneumoniae* infection is the most common cause of bacterial pneumonia, especially in low–middle-income countries (LMIC) that lack effective immunization schedules and policies. Pneumococcal pneumonia is transmitted via droplets or aerosols, with nasopharyngeal colonization being a prerequisite for infection. The carriage of *S. pneumoniae* is highest among populations aged 2–3 years and dwindles beyond that age to <10% in adults. Pneumococcal pneumonia is usually characterized by the lobar form of lung injury, typically with no long-term negative effect on the lungs. Globally *S. pneumoniae* infections account for about 2 million deaths per year, and the incidence is more pronounced at the extremes of age. Additionally, the presence of comorbidities and immune incompetence augments the incidence and morbidity associated with pneumococcal pneumonia [114,115]. Severe forms of pneumococcal pneumonia can result in ARDS and other pulmonary implications such as abscesses, empyema, multilobar infiltration, and pleural effusion. These complications are associated with increased morbidity and the development of septic shock [116]. Interestingly, bacteremia associated with *S. pneumoniae* is documented in about 20–25% of cases, with an increased risk and poorer outcome among high-risk groups. These high-risk groups include immunocompromised individuals like the aged and HIV patients [117]. It is worth mentioning that bacterial cultures are positive for approximately 50% of sepsis cases; thus, culture-negative pneumococcal infection with sepsis can be a common diagnostic outcome. The blood culture of patients with extrapulmonary pneumococcal infection and severe sepsis would yield *S. pneumoniae* predominantly and/or well-known sepsis-related bacteria such as *K. pneumoniae*, *S. aureus*, *Legionella pneumophila*, *E. coli*, *H. influenzae*, *E. cloacae*, *K. oxytoca*, *Veillonella* spp., and *Enterococcus* spp. [3].

The development of pneumococcal pneumonia is associated with risk factors involving the interplay of pathogen characteristics (e.g., virulence) and host susceptibility. Pathogen factors that influence the development and severity of pneumococcal pneumonia include serotypes [118] and the impact of initial antimicrobial treatment on bacteria [119,120]. Host characteristics that are considered risk factors for the development and severity of pneumococcal pneumonia are age, gender (males are at an increased risk), tumors, unhealthy lifestyle/habits (alcoholism and smoking), immunosuppression (e.g., HIV), and hepatic and renal complications. Additionally, vaccination (immunization status) and host genetic polymorphisms are important risk factors for pneumococcal pneumonia [121,122,123]. 

Mortality is alarmingly high among patients with pneumonia, complicated by septic shock [4]. A study carried out in Spain identified septic shock as a frequent complication of pneumococcal pneumonia, with approximately 11% of patients presenting with septic shock on admission [5]. In summary, pneumococcal pneumonia is considered a risk factor for critical health vulnerability similar to long-term mortality and morbidity of chronic diseases, like cardiovascular disease and stroke.

## 5. The Pneumococcus and Septic Inflammation

### 5.1. Pneumococcus and the Innate Immune System

The body’s immediate defense to insult and injury, known as the innate immune system, is vital for protection in the early stages of infections, including pulmonary infections. This line of defense includes a myriad of immune cells, mediators, and mechanisms that identify and resist the establishment of infections in a nonspecific fashion [124]. The lungs are vital organs for vertebrates’ gaseous exchange and constantly interact with our external environment. Breathing predisposes the lung tissue to risk of infection and disease from pathogenic microorganisms in the environment, such as airborne pathogens, toxic pollutants, and allergens [125]. The primary defense mechanism against infection and inflammation from these agents involves the successful identification of the offending agent, eliciting the appropriate immune response and the containment of bacteria growth in the lung. Microbial growth containment ensures that infectious agents such as bacteria are not disseminated to cause systemic inflammation. *Streptococcus pneumoniae* is the main pathogen implicated in CAP, a life-threatening lung infection of global impact. Inhalation of *S. pneumoniae* into the lungs imitates the activation of lung-resident innate cells, which begins the complex pathogenesis of pneumonia. Innate immune cells in the lungs—chiefly, specialized macrophages (alveolar macrophages), neutrophils, dendritic cells, and natural killer cells—can recognize components of the bacteria known as pathogen-associated molecular patterns (PAMPs) through pattern-recognition receptors (PRRs) expressed on their membrane surfaces [126]. Epithelial cells of the airways comprising bronchial epithelial cells and alveolar epithelial cells also serve as innate immune cells aside from their primary function in gaseous exchange. A specialized type of airway epithelial cells (AEC), known as type II AEC or pneumocytes, serves as a mechanical barrier in the lungs to protect against *S. pneumoniae.* During pneumonia, this mechanism of defense is achieved via their ability to recognize PAMPs of pneumonia-causing pathogens through their PRPs [a repertoire of Toll-like receptors (TLRs) and NOD-like receptors (NLRs)]. In addition to their ability to interact with other immune cells, type II AECs secrete antimicrobial peptides, cytokines, surfactant proteins, and mucus to initiate and complement the mechanism of pathogen clearance [127,128]. Phagocytes such as neutrophils are usually the first to infiltrate the site of infection in large quantities. Neutrophils engulf bacteria cells such as *S. pneumoniae* and ultimately kill them by the release of granules such as defensins and degrading enzymes. Neutrophils can also trap *S. pneumoniae* extracellularly via extracellular fibers composed of nucleic acids [128,129]. The macrophages are another class of phagocytes that also engulf and directly kill *S. pneumoniae*. In the presence of pneumococcus TLR4 and TLR2 complementarily activate macrophages that chemo-attract neutrophils by cytokine signaling to mob up dead neutrophils and other cells as well as antigens [130]. The pneumococcal polysaccharide capsule is recognized by PRPs expressed on macrophages. The C-type lectin known as SIGN-R1 expressed by macrophages binds capsular polysaccharide and/or the whole pneumococcal cell [130]. 

### 5.2. Cross-Talk between Pneumococcus and Dendritic Cell

The major immune cells pivoting the innate and adaptive immune response are the dendritic cells (DCs). DCs are sentinel professional phagocytes that play a curial role known as antigen processing and presentation; thus, they are specialized immune cells that collect (ingest) and process pathogens and antigens to be recognized and presented to effector cells of the adaptive immune system. This demonstrates DCs’ important function as the ‘connector’ of the innate and adaptive immune response. DCs include several classes and subpopulations that are phenotypically and functionally diverse [131,132]. Although the mechanisms underlying the recognition, ingestion, and intracellular degradation of *S. pneumoniae* by DCs have not been extensively studied, the general role of DCs in ameliorating pulmonary pathogenic infections is known. Generally, pulmonary DCs (CD103+ PDCs) play an important role in the regulation of T helper 2 cells (Th2) and Th17 immunity. Distinct populations of lymph node DCs (CD11c+ DCs) have been identified to express the C-type lectin receptor, SIGN-R1+, and thus bind *S. pneumoniae* for opsonization with complement proteins and immunoglobulins [133]. The recognition and binding of the pneumococcal polysaccharide capsule by SIGN-R1 activate the classical complement pathway via C1q to allow for the opsonization and deposition of pneumococcal antigens on follicular dendritic cells (FDCs). This process is very necessary to initiate a humoral immune response specifically against *S. pneumoniae* [134].

### 5.3. Potential Role of Dendritic Cells in Extrapulmonary Dissemination of Bacteria

**Figure 1:**
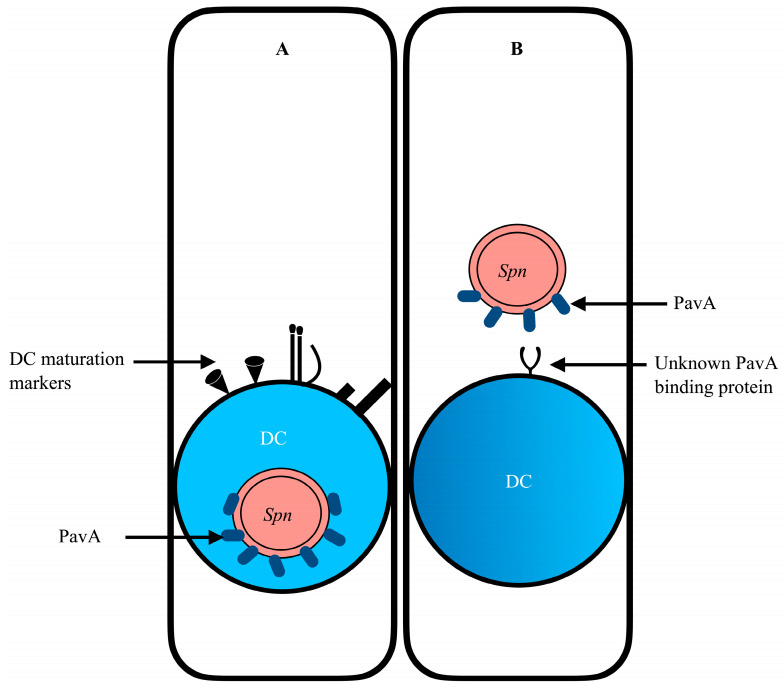
The potential role of PavA in immune evasion and extrapulmonary dissemination of *S. pneumoniae*. (**A**) The expression of PavA on internalized pneumococcus enhances the maturation of DCs and, therefore, reduces phagocytic potency—a potential mechanism to impede the uptake of *S. pneumoniae* (*Spn*). (**B**) The possibility of PavA binding an unknown surface protein on immature DCs to elicit downstream signaling events that aid in immune evasion and bacterial dissemination.

Pulmonary DCs, in conjunction with AECs, form a synergistic network in pathogen recognition. Upon encountering pathogens, DCs in the airways mature and migrate to the T cell zone of the draining lymph nodes. DCs in their immature state efficiently phagocytose or macropinocytose bacteria and process them into host-recognizable antigens. However, DCs readily mature upon pathogen encounter into professional antigen-presenting cells (APCs), expressing maturation proteins such as co-stimulatory molecules (CD80, CD86, CD40) and major histocompatibility complexes (MHC) [135]. While DC maturation is necessary for the optimal priming of naive T cells, it diminishes the capacity for internalization and antigen processing (Figure 1). There is evidence that some microbial pathogens harness the migratory capacity of DCs as vehicles of systemic dissemination, thus assuming a “Trojan horse” trait [136,137]. Recent studies support the strong evidence that *S. pneumoniae* may potentially exploit the capacity and function of DCs to facilitate the dissemination of bacteria from primary infection sites. Notably, *S. pneumoniae* possesses virulent factors such as pneumococcal adherence and virulence factor A (PavA) that dampen the phagocytic and antigen-processing efforts of DCs (Figure 1). The expression of PavA in *S. pneumoniae* has been found to impede the uptake of the bacteria by DCs in vitro [138]. What remains unclear, however, is the specific role of DCs in pneumococcal infection and extrapulmonary dissemination. PavA is known to bind the extracellular matrix protein (ECMp) fibronectin [139] for adhesion; however, the direct mechanism/signaling pathways that pneumococcal PavA employs to impede uptake by DCs to influence bacteria dissemination remain to be elucidated (Figure 1).

Sepsis, a potential complication of extrapulmonary bacteria dissemination, influences the role of DCs in immune responses. Notably, DCs undergo pathological changes induced by sepsis via several alteration mechanisms like ROS generation, epigenetic regulation, and induction of apoptosis. These alterations include changes in the number of various DC subsets, differentiation, and levels of surface functional markers [140]. Such alterations have diagnostic and therapeutic implications. For example, septic DCs have been shown to exhibit aberrant cytokine secretion, playing a role in sepsis-induced immunoparalysis [141]. Sepsis augments the population of splenic DCs that do not express CD8 and CD4 molecules; however, it induces a substantial loss of CD8- or CD4-expressing splenic DCs [142]. Additionally, the spatiotemporal role of septic DCs reported by some studies suggests that systemic- and mucosal-derived subsets of DCs impact the proliferation of CD4 T cells differently and ultimately influence disease patterns in sepsis. Reports from murine-modeled experiments suggest that septic mucosal DCs have a relatively high expression of surface activation markers such as MHC-II and CD 40 compared to their systemic counterparts [131]. Furthermore, septic DCs expressing CD1a+ patients tend to induce the expansion of T cells that are characterized by the possession of tolerance or regulation maker (FoxP3) and, hence, exhibit stronger regulatory function [143]. In the absence of DCs, there seems to be an improved resistance to *S. pneumoniae* infection in mice. This observation results from a significant reduction in the dissemination of the bacteria from the lungs to the lymph nodes, following the depletion of DCs [144]. Additionally, DC-depleted mice were found to express relatively low levels of systemic inflammatory cytokines compared with mice with intact DCs after intranasal inoculation with *S. pneumoniae.* Despite the lower systemic inflammation observed in the DC-depleted mice, the recruitment of leukocytes remained comparable in the presence or absence of DCs in mice intra-nasally inoculated with *S. pneumoniae* [144]. Considering DCs’ pivotal role in immune activation and septic inflammation, modifications to the maturation, function, population, distribution, and survival of DC subsets in sepsis need much attention in our quest for effective immunotherapy strategies. 

### 5.4. Interleukin 37 (IL-37) and Severe S. pneumoniae Infections

*S. pneumoniae* infections can be severe and complicated, characterized by heightened immune activation and excessive inflammatory responses resulting in tissue damage. A high bacteria burden and the risk of systemic bacterial spread are two important factors that influence the severity of *S. pneumonia* infections [145]. Thus, a crucial objective in host defense against pneumococcal pneumonia is the elimination of *S. pneumoniae* from the lungs and the prevention of extrapulmonary dissemination. *IL-37*, a member of the *IL-1* family of cytokines and a known fundamental anti-inflammatory/immunosuppressive cytokine, has gained attention for its potential role in exacerbating chronic infections and the risk for severe *S. pneumoniae* disease [146]. The impact of *IL-37* on pneumococcal infections is more debilitating than advantageous to resolving inflammation, as recent findings implicate the cytokine as an inhibitor of innate immune response [147]. Murine studies have shown that the expression of *IL-37* in tissue impedes the control of pneumococcal growth and, consequently, augments inflammation. Thus, the expression of *IL-37* expression in alveolar macrophages decreases their capacity to intracellularly kill pneumococci *in vitro*. The excessive immune activation and inflammation as a result of enhanced intracellular survival of bacteria leads to an increased influx of inflammatory mediators, intensified tissue damage, necrosis, and risk of bacterial dissemination [148].

## 6. Conclusions

Pneumonia and sepsis are two important clinical syndromes with a significant global burden, both of which are characterised by complex pathophysiology. Pneumonia is a common complication among critically ill patients including patients with severe sepsis. *Streptococcus pneumoniae* is a key pathogen implicated in both sepsis and pneumonia. Growing evidence suggests that pneumococcal pneumonia can potentially disseminate and consequently induce systemic inflammation and severe sepsis. Additionally, immune mediators such as the immunosuppressive cytokine *IL-37* contribute to the severity and extrapulmonary spread of *S. pneumoniae* to potentially induce sepsis and systemic complications. In summary, we have discussed how sepsis-induced immune compromise may facilitate the establishment of secondary infections such as infections from *S. pneumoniae.* This is supported by the evidence that the height of immunosuppression induced by severe sepsis correlates with the occurrence and severity of pneumonia. Increased susceptibility to nosocomial pneumonia in children with multiple organ dysfunction syndrome is associated with the persistence of immune paralysis. *S. pneumoniae* may potentially hijack the function of DCs via PavA to spread from the lung (local site of infection) and elicit systemic inflammation that may result in severe sepsis. More research into the immunologic crosstalk between pneumococcus and host immune mediators is needed to fully understand the pathophysiology of pneumonia-induced lung injury and severe sepsis.
